# Stable and Ultrafast Blue Cavity‐Enhanced Superfluorescence in Mixed Halide Perovskites

**DOI:** 10.1002/advs.202301589

**Published:** 2023-05-01

**Authors:** Linqi Chen, Danqun Mao, Yingjie Hu, Hongxing Dong, Yichi Zhong, Wei Xie, Nanli Mou, Xinjie Li, Long Zhang

**Affiliations:** ^1^ Key Laboratory of Materials for High‐Power Laser Shanghai Institute of Optics and Fine Mechanics Chinese Academy of Sciences Shanghai 201800 China; ^2^ Center of Materials Science and Optoelectronics Engineering University of Chinese Academy of Sciences Beijing 100049 China; ^3^ State Key Laboratory of Precision Spectroscopy School of Physics and Electronic Science East China Normal University Shanghai 200241 China; ^4^ Key Laboratory of Advanced Functional Materials of Nanjing Nanjing Xiaozhuang University Nanjing 211171 China; ^5^ Hangzhou Institute for Advanced Study University of Chinese Academy of Sciences No.1, Sub‐Lane Xiangshan, Hangzhou Xihu 310024 China; ^6^ CAS Center for Excellence in Ultra‐intense Laser Science Shanghai 201800 China

**Keywords:** blue cavity‐enhanced superfluorescence, halide phase segregation, perovskite, quantum dots, superlattice microcavities

## Abstract

Cavity‐enhanced superfluorescence (CESF) in quantum dot (QD) system is an ultrafast and intense lasing generated by combination of quantum coupling effect and optically stimulated amplification effect, which can provide a new idea for realizing high quality blue light sources and address the limitation of conventional inefficient blue light sources. Modifying halide composition is a straightforward method to achieve blue emission in perovskite QD system. However, the spectral instability introduced by photoinduced halide phase segregation and low coupling efficiency between QDs and optical cavities make it challenging to achieve stable blue CESF in such halide‐doped QD system. Herein, long‐range‐ordered, densely packed CsPbBr_2_Cl QD‐assembled superlattice microcavities in which the two core issues can be appropriately addressed are developed. The QD superlattice structure facilitates excitonic delocalization to decrease exciton‐phonon coupling, thus alleviating photoinduced phase segregation. By combination of theoretical analysis and temperature‐dependent photoluminescence (PL) measurements, the underlying photoinduced phase segregation mitigation mechanism in mixed halide superlattices is clarified. Based on the CsPbBr_2_Cl QD superlattices with regularly geometrical structures, in which the gain medium can be strongly coupled to the naturally formed microcavity, stable and ultrafast (3 ps) blue CESF with excellent optical performance (threshold ≈33 µJ cm^−2^, quality factor ≈1900) is realized.

## Graphical abstract

Photoinduced phase segregation and low coupling efficiency between QDs and cavities make is challenging to achieve stable blue cavity‐enhanced superfluorescence in halide‐doped perovskite QD system. Here, long‐range‐ordered CsPbBr_2_Cl QD superlattices are developed, in which the two core issues can be appropriately addressed. Based on the CsPbBr_2_Cl QD superlattices with regularly geometrical structures, stable, and ultrafast blue cavity‐enhanced superfluorescence is realized.

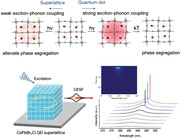

## 1 Introduction

Superfluorescence (SF) is a unique quantum optical phenomenon generated by cooperatively coupled emitters that manifests as a short and intense burst of light. Owing to the unique intrinsic physical mechanism and promising prospect for various photonic applications, including lasing, on‐chip integration in optical computing,^[^ ^1^, ^2^, ^3^ ^]^ and ultrafast biosensing, SF has garnered considerable research interest.^[^ ^4^, ^5^, ^6^, ^7^, ^8^, ^9^, ^10^ ^]^ Recently, trihalide perovskite quantum dot (QD) system with large oscillator strength, high density of exciton states, and long dephasing time was reported to be an excellent platform for SF research.^[^ ^11^, ^12^, ^13^, ^14^, ^15^, ^16^, ^17^, ^18^ ^]^ If such a many‐body quantum ensemble is coupled well with optical cavity, the photons will pass through the system repeatedly before leaking out of the cavity owing to the feedback effect of cavity. Consequently, the interaction intensity between light and matter significantly increases, thereby, resulting in cavity‐enhanced superfluorescence (CESF), which helps to further reduce the concentration threshold of collective radiation and increase the radiation intensity.^[^ ^12^, ^19^ ^]^ It combines the characteristics of stimulated resonance emission and the collective effect of SF radiation.^[^ ^20^ ^]^ The outstanding performance of CESF is expected to be applied to blue light emission and help address the limitation of inefficient blue light sources. However, it is still a great challenge to realize blue CESF in perovskite QD system owing to lacking suitable gain medium and inefficient coupling between gain material and microcavity.

A direct method for realizing blue emission in perovskite QD system is halide component engineering. Mixed halide (Br and Cl) perovskite QDs can sufficiently perform bandgap tailoring and cover the entire blue spectrum range.^[^ ^21^, ^22^, ^23^, ^24^, ^25^, ^26^ ^]^ However, the realization of stable blue CESF in such mixed halide perovskite QD system is limited by two major factors: the spectral instability caused by photoinduced halide phase segregation^[^ ^27^, ^28^ ^]^ and inefficient coupling of QD medium with optical cavities.^[^ ^20^, ^29^, ^30^ ^]^ Owing to the low halogen migration energy barrier, inevitable photoinduced ion migration and halide phase segregation in mixed halide QDs restrict their stability and hinder their further development.^[^ ^31^, ^32^, ^33^, ^34^, ^35^, ^36^ ^]^ To address this issue, considerable efforts have been devoted to elucidate the underlying mechanism of photoinduced phase segregation, and significant analysis results have been published. The charges deform the surrounding lattice via electron‐phonon coupling, and then, the lattice strain promotes halide phase segregation.^[^ ^37^, ^38^, ^39^, ^40^ ^]^ Hence, reducing electron‐phonon coupling is a theoretically effective approach to alleviate phase segregation. It may be a feasible strategy to assembly mixed halide QDs into QD superlattices since the enhanced excitonic delocalization effect of QD superlattice would contribute to reducing exciton‐phonon (EP) coupling.^[^ ^37^, ^41^ ^]^ Meanwhile, the long‐range‐ordered, densely packed superlattices with smooth surface and regular 3D structures can simultaneously serve as gain medium and whispering gallery cavity with highly optical feedback, which is beneficial for addressing the coupling efficiency issue of QDs with optical cavities. Therefore, the two core issues mentioned previously can be efficiently addressed in QD self‐assembled superlattice structures.

In this work, we successfully synthesized well‐defined CsPbBr_2_Cl QDs self‐assembled superlattices and demonstrated CsPbBr_2_Cl superlattices in which the gain medium facilitates excitonic delocalization to decrease EP coupling can mitigate photoinduced phase segregation. The underlying photoinduced phase segregation alleviation mechanism in superlattices was elucidated by combining theoretical analysis and temperature‐dependent photoluminescence (PL) measurements. Meanwhile, the obtained CsPbBr_2_Cl QD superlattices with regular geometry can simultaneously serve as gain medium and optical cavity. We further realized stable and ultrafast (3 ps) blue CSEF with excellent optical performance (threshold ≈33 µJ cm^−2^, quality factor ≈1900) by integrating cooperative excitonic ensemble coupling in CsPbBr_2_Cl QD superlattice with optical cavity coupling. Additionally, the ultrafast optical property and dynamic process of blue CESF were analyzed by time‐resolved photoluminescence (TRPL).

## 2 Results and Discussion

**Figure 1:**
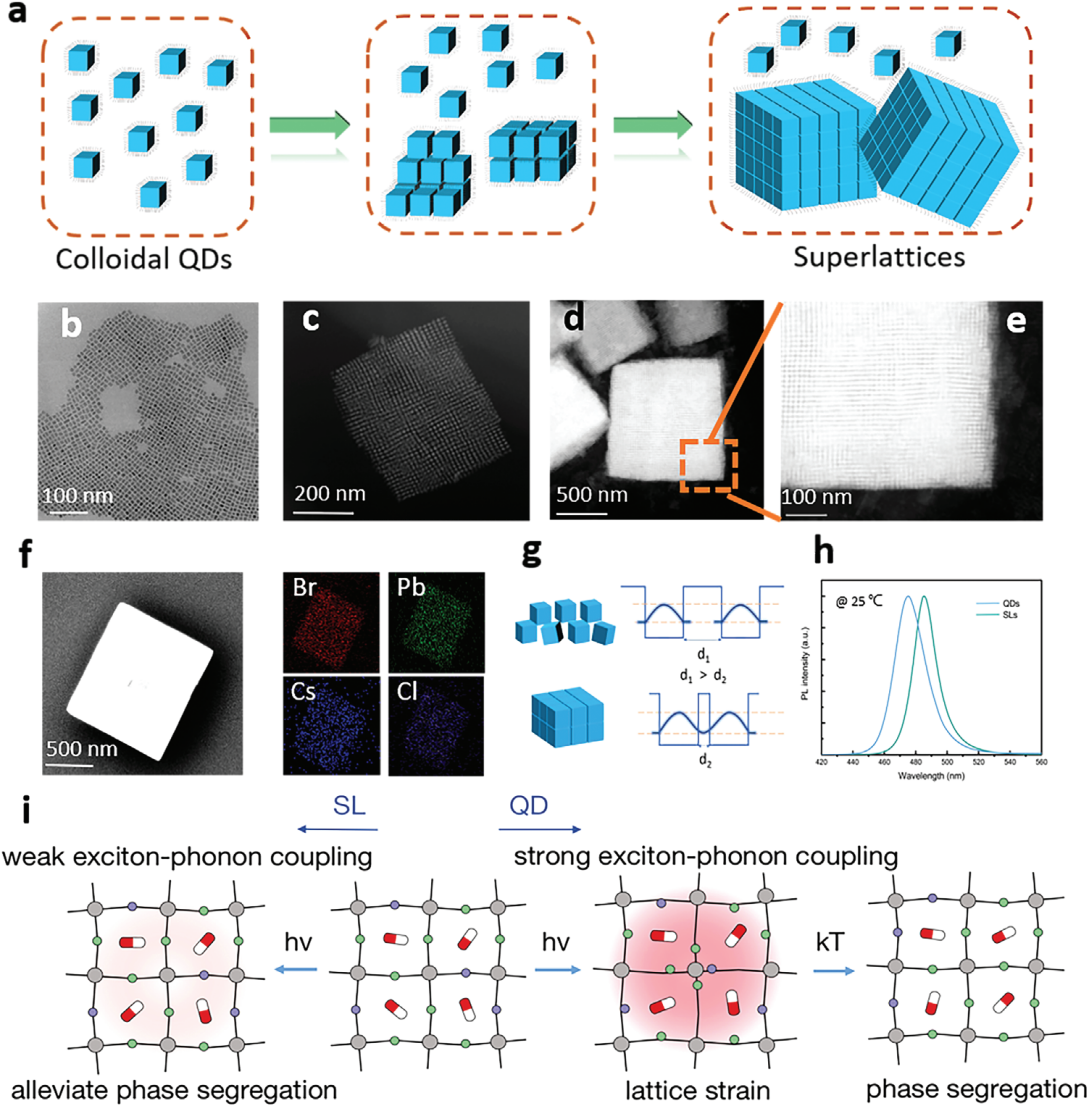
Preparation and structural and optical characterization of superlattices. a) Schematic of the superlattice formation process. b) TEM image of monodispersed CsPbBr_2_Cl QDs. c) TEM image of superlattice clusters. d) TEM image of the regularly shaped superlattice cube. e) Magnified view of (d). f) EDXS reveals the elemental composition of the self‐assembled superlattice. g) Schematic diagram of delocalization of the carrier wavefunction in superlattices. h) PL spectra of uncoupled QDs and SLs. i) Schematic of CsPbBr_2_Cl SLs mitigating the photoinduced phase segregation through weakening exciton‐phonon coupling compared to uncoupled QDs.

CsPbBr_2_Cl QDs were prepared as self‐assembly blocks, following a previously reported method with modifications, and the synthesis details are presented in the experimental section. The dispersed CsPbBr_2_Cl QDs in a toluene solution can slowly gather together under the interaction of the weak molecular force to form long‐range ordered QD superlattices with regular geometry. Simultaneously, low‐temperature environmental conditions enable a slower and more orderly self‐assembly process. Herein, the self‐assembly temperature of CsPbBr_2_Cl QDs was set at ≈12 °C. The colloidal CsPbBr_2_Cl QD solution in a brown reagent flask was aged for a period of time at ≈12 °C, and precipitation was observed at the bottom of the reagent flask, implying the formation of assemblies. **Figure** **1**a comprehensively depicts the self‐assembly evolution process from monodisperse colloidal QDs to closely packed and highly symmetric QD superlattices through intermolecular interactions between ligands of the QD surface. Additionally, the morphology and monodispersity of QDs play a vital role in the formation of ordered assemblies. The monodispersity of QDs can be further improved by optimizing the centrifugal purification separation process during QDs preparation, the details of which are presented in the experimental section. Figure 1b shows a typical transmission electron microscopy (TEM) image of the as‐prepared CsPbBr_2_Cl QDs, indicating that the product consists of uniform and nearly ideal nanocubes with side length of ≈ 8 ± 2 nm. The investigated CsPbBr_2_Cl QDs possess the 3D‐perovskite orthorhombic crystal structure (Pnma space group), shown in Figure S1 (Supporting Information). Theoretical X‐ray diffraction (XRD) pattern of orthorhombic CsPbBr_2_Cl is shown in Figure S2 (Supporting Information), consistent with the experimental XRD result (Figure S3, Supporting Information). As revealed by TEM, the monodispersed QDs self‐assemble as they slowly age in solution to form cubic superlattices (Figure 1c–e). Additionally, selected area electron diffraction were conducted on the superlattice structure and QD clusters with lower degree order respectively, as shown in Figure S4a,c (Supporting Information). The corresponding patterns are shown in Figure S4b,d (Supporting Information), which can further confirm the periodic ordered arrangement structure of the superlattice. The superlattice shows high packing factor and sharp edge, facilitating optical feedback necessary for lasing. And large area TEM image of superlattices is shown in Figure S5 (Supporting Information). The elemental composition of the self‐assembled QD superlattices was confirmed by the Energy‐dispersive X‐ray spectroscopy (EDXS) shown in Figure 1f, and Cs, Pb, Br, and Cl elements present uniform spatial distribution. As the QDs self‐assemble into superlattices, the decreased interdot distance leads to a spatial distribution of electrons and holes and be mapped as the wavefunctions of neighboring QDs overlap as illustrated in Figure 1g. And as the coupling between QDs increases at shorter interdot distances, the electronic states split to form bands, which eventually results in the lower PL peak energy of superlattices.^[^ ^42^, ^43^, ^44^ ^]^ The increase in carrier delocalization can be verified by PL spectra comparison of QDs and superlattices as shown in Figure 1h. The PL emission of QDs is centered ≈ 475 nm. Compared to isolated QDs, the emission peak of the superlattices redshifts to ≈ 485 nm, which originates from the overlapping of individual QD wavefunctions in the superlattice structures. Additionally, QDs of the same size have priority in the self‐assembly process, thus narrowing the emission line width.

The soft and dynamic lattice properties of metal halide perovskite make it sensitive to external stimulation. The main inducement of photoinduced phase segregation in such halide‐doped perovskites is continuous light illumination and strong electron‐phonon coupling, which promote the formation of internal polaron, then induce lattice strain and reduce the energy barrier of ion migration.^[^ ^37^, ^38^, ^39^, ^40^ ^]^ The corresponding density functional theory (DFT) simulations are shown in Figure S6 (Supporting Information). Figure S6a (Supporting Information) shows the lattice strain after the generation of polaron, consistent with expansive strain. Lattice strain can reduce the energy barrier of halogen anion migration, thus promoting halide phase segregation. As shown in Figure S6b (Supporting Information), the anion migration energy barrier decreases with the increase of lattice strain, further accelerating the halide phase segregation. The polarization of the lattice can be reduced by decreasing the electron‐phonon coupling, thereby helping to reduce the formation of polarons and thus inhibiting photoinduced phase segregation.^[^ ^45^ ^]^ Theoretically, the electron wavefunction coupling in the superlattice facilitates excitonic delocalization and thus can reduce the EP coupling in contrast to uncoupled QDs under irradiation.^[^ ^46^ ^]^ The underlying photoinduced phase segregation mitigation mechanism in CsPbBr_2_Cl superlattice is illustrated in Figure 1i.

**Figure 2:**
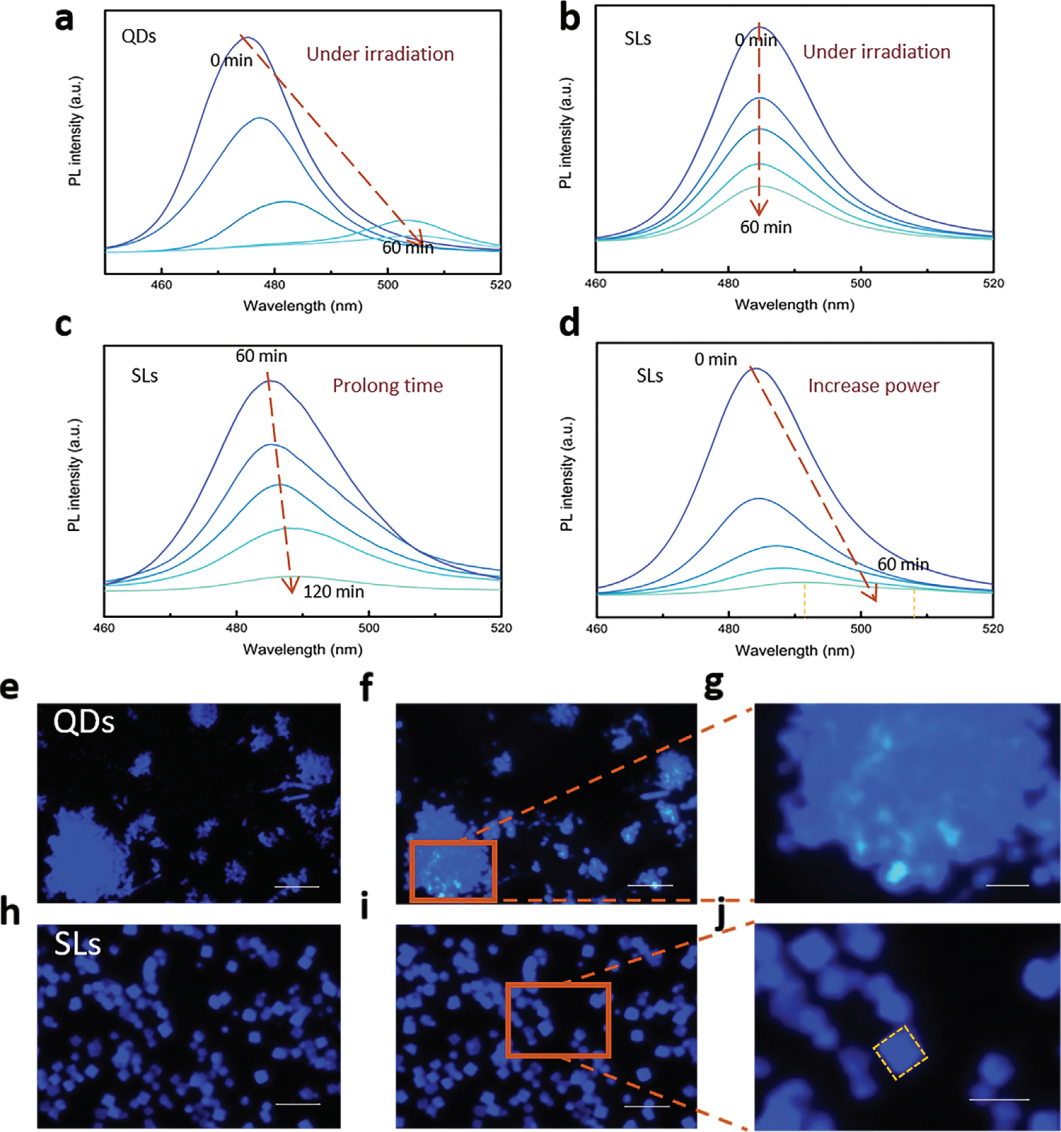
CsPbBr_2_Cl QDs self‐assembled superlattices (SLs) alleviating photoinduced phase segregation. a) Time‐dependent PL spectra evolution of CsPbBr_2_Cl QDs under CW laser radiation (78 W cm^−2^). b) Time‐dependent PL spectra evolution of CsPbBr_2_Cl SLs under CW laser radiation (0–60 min). c) Time‐dependent PL spectra evolution of CsPbBr_2_Cl SLs under CW laser radiation (60–120 min). d) Time‐dependent PL spectra evolution of CsPbBr_2_Cl SLs under CW laser radiation with increasing power (147 W cm^−2^). e) PL images of CsPbBr_2_Cl QDs before light soaking. Scale bar, 5 µm. f) PL images of CsPbBr_2_Cl QDs after light soaking. Scale bar, 5 µm. g) magnified view of (f). Scale bar, 1 µm. h) PL images of CsPbBr_2_Cl SLs before light soaking. Scale bar, 5 µm. i) PL images of CsPbBr_2_Cl SLs after light soaking. Scale bar, 5 µm. j) magnified view of (i). Scale bar, 2 µm.

Experimentally, as expected, the CsPbBr_2_Cl superlattices exhibit better PL spectral stability than CsPbBr_2_Cl QDs under continuous laser irradiation (405 nm continuous‐wave (CW) laser). After light soaking, significant halide phase segregation of the QDs sample is observed through PL images under 50 × microscope (**Figure** **2**e,f). Additionally, the optical microscopy image of CsPbBr_2_Cl superlattices is presented in Figure S7 (Supporting Information). Figure 2g is the magnified view of marked part of Figure 2f, showing the presence of Br‐rich green emission points in blue emission area. In contrast, the square superlattice samples in Figure 2h maintain halogen uniformity after a period of light soaking (Figures 2i,j). It should be noted that when obtaining PL images after irradiation, the excitation laser intensity was adjusted, so that the samples appear brighter for obtaining clearer results. It is well known that long time irradiation could lead to the PL attenuation or quenching of samples to a certain extend. Furthermore, halide phase segregation can also be directly observed from PL spectral changes. The time‐dependent PL spectra of QDs and superlattices under laser source irradiation are presented in Figures 2a,b. Under CW laser irradiation of 78 W cm^−2^, the PL intensity of QDs decreases gradually with time, and the peak at ≈475 nm is gradually replaced by the peak at ≈505 nm. Contrastively, the PL peak position of the superlattice sample remains unchanged, and only the PL intensity decreases within 60 min. It can be concluded that the superlattice structure has better immunity to photoinduced phase segregation. Further prolonging irradiation time to 120 min (Figure 2c), the CsPbBr_2_Cl superlattices only show slight redshift. And increasing the irradiation power to 147 W cm^−2^, we can see that the PL intensity of the superlattices declines faster over time and finally the PL spectrum exhibits weak double peaks after 60 min, indicating slight halide phase segregation (Figure 2d; Figure S8, Supporting Information). The experimental results indicate that QDs self‐assembled superlattice structures can effectively alleviate the photoinduced halide phase segregation phenomenon compared to uncoupled QDs, which is consistent with our hypothesis.

**Figure 3:**
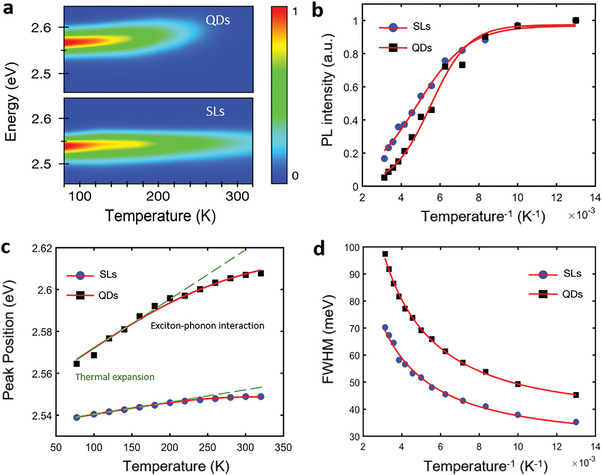
Temperature‐dependent PL characteristics of CsPbBr_2_Cl QDs and SLs. a) Contour plots of the temperature‐dependent PL from QDs and SLs with the temperature variations from 77 to 320 K. Evolution of b) PL intensity, c) PL peak energy, and d) PL spectral FWHM for QDs and SLs with increasing temperature.

To further verify the theoretical analysis of the mitigation of photoinduced halide phase segregation, we performed temperature‐dependent PL measurements, which can reflect the strength of EP coupling. Temperature‐dependent PL spectra of the QDs and superlattices are shown in **Figure** **3**a. Significantly, the optical properties of superlattices are better than those of uncoupled QDs. And as shown in Figure 3b, the temperature‐dependent PL intensity of QDs and superlattices can be fitted by the Arrhenius equation,

(1) $$I\left(T\right)=\frac{I\left(0\right)}{1+Ae^{-E_{b}/k_{B}T}}$$

where I(0) is the intensity at low temperature, E_b_ is the exciton binding energy, A is a constant and k_B_ is the Boltzmann constant. The fitting E_b_ of superlattices is smaller than that of QDs. EP coupling is associated with high exciton binding energy because of enhanced Coulomb electron‐hole interactions. The exciton binding energy of superlattices (105.1 meV) is reduced by 34% compared to that of uncoupled QDs (141.3 meV), which also implies weaker EP coupling. Figure 3c shows the decreasing blueshifts of PL peak energies with increasing temperature for superlattices and QDs owing to the thermal expansion (TE) and EP interactions.^[^ ^47^, ^48^ ^]^ The TE and EP interactions determine the temperature‐dependent evolution of peak energy by the following equation:

(2) $$E_{g}\left(T\right)=E_{0}+A_{T}T-A_{EP}\left(\frac{2}{\exp\left(\hbar \omega /k_{B}T\right)-1}+1\right)$$

in which E_0_ is the unrenormalized bandgap, ℏ*ω* is the average optical phonon energy, A_T_ and A_EP_ represent the weight of TE and EP interaction, respectively. When the TE contribution is solely considered (green dashed line in Figure 3c), the linear blueshift of peak positions is dominantly observed. The EP coupling is negligible at low temperature owing to the unsubstantial population of optical phonons. As the temperature increases, the EP interaction contributes negatively to the PL peak energy, and the A_EP_ of superlattices is calculated to be smaller than that of QDs. The corresponding fitting parameters are shown in Table S1 (Supporting Information). Moreover, as temperature increases, the full width at half maximum (FWHM) of the PL peak is significantly influenced by the interactions between excitons and thermally induced phonons, which decrease the radiative efficiency of excitons.^[^ ^49^ ^]^ This suggests that the EP coupling strength contrast can also be revealed by FWHM as a function of temperature. In fact, the FWHM is closely related with the inhomogeneity of QD size and the interactions between excitons and phonons.^[^ ^50^ ^]^ The experimental data of PL broadening are fitted well with

(3) $$\Gamma \left(T\right)=\Gamma_{\mathrm{inh}}+\Gamma_{\mathrm{AC}}T+\Gamma_{\mathrm{LO}}{\left(e^{E_{\mathrm{LO}}/k_{B}T}-1\right)}^{-1}$$

(Figure 3d), where *Г*_inh_ represents the temperature‐independent intrinsic inhomogeneous linewidth. *Г*_AC_ is the coefficient of the acoustic phonon‐exciton interaction, *Г*_LO_ is the longitudinal optical (LO) phonon‐exciton coupling coefficient, and *E*_LO_ is the LO‐phonon energy. The fitting parameters are summarized in Table S2 (Supporting Information). The *Г*_inh_ (28.4 meV) of superlattices is smaller than that (36.9 meV) of QDs, which indicates that the size uniformity of QDs in superlattice samples is higher. At the same time, the *Г*_AC_ (81.0 µeV K^−1^) and *Г*_LO_ (30.6 meV) of QDs are both greater than those (*Г*_AC_ = 50.2 µeV K^−1^, *Г*_LO_ = 15.5 meV) of superlattices. We thus can conclude that the superlattice structures can suppress the EP interaction, which induces polaron and dominates lattice strain. To sum up, compared with uncoupled QDs, the experiment results show that superlattice structures can ease EP coupling and thereby reduce photoinduced phase segregation effect.

**Figure 4:**
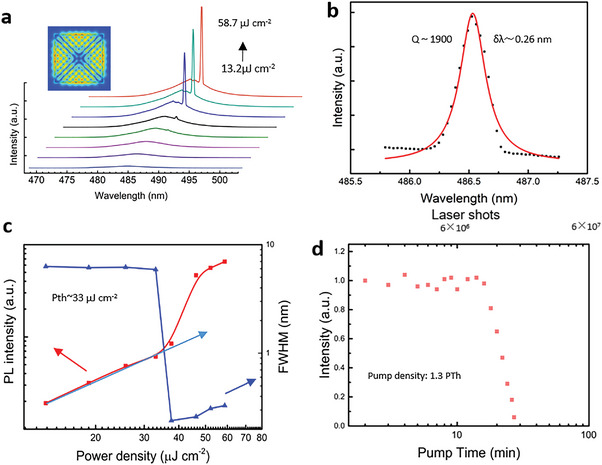
Blue CESF in CsPbBr_2_Cl QD superlattices. a) Power‐dependent emission spectra of a typical CsPbBr_2_Cl superlattice. Inset: 2D optical WGM distribution. b) Lorentz fitting of a lasing peak with a FWHM (*δλ*) of ≈0.26 nm, corresponding to a Q factor of ≈1900. c) PL intensity and spectral FWHM as functions of pumping density. d) Real‐time‐integrated emission intensity variation with pumping time, the stable intense output can be maintained for ≈30 min.

Furthermore, based on such excellent CsPbBr_2_Cl superlattice structures with high gain packing density and coupling efficiency, we realized blue CESF at ≈486.5 nm as shown in **Figure** **4**a. A self‐built confocal microscope PL system (Figure S9, Supporting Information) was used to measure the power‐dependent PL spectra at 77 K with a 400 nm femtosecond laser as the excitation source. The PL spectra undergo a transition from broadband spontaneous radiation to stimulated radiation with a sharp peak as the pumping power increases. At low pumping density, a wider SF peak at 485 nm with a FWHM of 5.24 nm is observed (Figure S10, Supporting Information), which is significantly smaller than the FWHM of the PL spectrum of the CsPbBr_2_Cl perovskite QDs. As the excitation fluence increases, a unique small peak will appear on the SF background, indicating the achievement of CESF. Notably, a typical S‐shaped curve is observed in Figure 4c, which represents the nonlinear dependence of the PL intensity on the pumping density. Simultaneously, as the excitation density reaches the threshold of 33 µJ cm^−2^, CESF occurs and the FWHM plummets to 0.26 nm (*δλ*). And the lasing peak can be well fitted with the Lorentzian function as shown in Figure 4b, and the corresponding Q factor of ≈1900 can be calculated with Q = *λ*/*δλ*. In addition, the broadening of FWHM of the emission peak with increasing of pumping density can be noticed, which may be induced by excitation‐induced heating. The typical CESF performance of superlattices can be attributed to the great naturally formed whispering‐gallery mode cavity with high optical gain and the corresponding light field distribution simulation is shown in Figure S11 (Supporting Information). The Q factor obtained according to the simulation results is 2479.9, and the difference between the theoretical Q factor and the Q factor calculated by the experimental results is within a reasonable range. To analyze the stability of blue CESF, the samples were constantly pumped by a pulsed laser with a pumping density of 1.3 P_Th_. The real‐time‐integrated emission intensity varied with pumping time, as shown in Figure 4d. The stable intense output can be maintained for ≈30 min. In addition, the stability of QD superlattice structure is also a issue should be considered. In our experiment, the QD superlattice structure formed by slowly age and solvent evaporation induced self‐assembly on silicon wafer can be stably stored in the drying cabinet for one month. And the corresponding TEM images of the superlattices is shown in Figure S12 (Supporting Information), which still maintains a tightly ordered QD array structure, and the CESF can still be achieved at 77 K, as shown in Figure S13 (Supporting Information).

**Figure 5:**
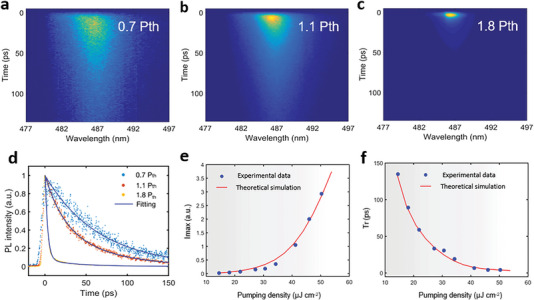
Dynamics analysis of blue CESF under different pumping densities. a) Streak camera image of SF at a low excitation density of 0.7 P_th_. b,c) Streak camera images of blue CESF at a high excitation density of 1.1 P_th_ and 1.8 P_th_, respectively. d) Typical TRPL decay curves under different pumping density. e) Peak intensity I_max_ of SF/CESF signals versus excitation density. The dots are experimental data and the line is theoretical simulation result. f) Power density dependence of radiative time T_r_.

The generation of CESF differs from that of traditional lasers, which originates from a cooperative exciton ensemble. Cooperative excitons exhibit quantum behavior during their lasing process, in which the perovskite superlattice system consumes all the cooperative components of dipoles by the CESF channel, instead of being limited by the population inversion, as in conventional lasers.^[^ ^12^ ^]^ Two pumping pulses with different time intervals and excitation fluence were used to explore the residual excitons after the fast‐radiation process. And a conventional microlaser of CsPbBr_2_Cl material was selected to perform a comparative experiment. The results in Figure S14 (Supporting Information) distinguish the characteristics of CESF and classical lasing, further confirm the generation of blue CESF in the CsPbBr_2_Cl superlattices. Furthermore, the ultrafast optical characteristics of blue CESF were investigated through time‐resolved PL (TRPL) measurements under different excitation densities. **Figure** **5**a–c shows the images of TRPL at varied power densities. P_th_ is the pumping density that can excite the sample transition from SF to CESF. It can be clearly seen that the PL intensity at resonance wavelength increases rapidly with the pumping density increasing from 0.7 P_th_ to 1.8 P_th_. Due to the strong contrast between resonant and non‐resonant TRPL signals, TRPL signals at resonant wavelength can completely suppress other signals, and only one spot can be observed under high pumping excitation. At a low pumping density of 0.7 P_th_, the time dynamics of PL can fit well with a single exponential function, which indicates that most excitons are coupled together and the slow process of spontaneous radiation by the dephased excitons can be ignored (Figure 5d). And bi‐exponential fitting is necessary for superlattice samples with more defects or low exciton coupling efficiency. The fast component is related to the collective emission of coupled excitons and the slow component originates from spontaneous radiation of dephased excitons. As the pump power reaches the threshold, the lifetime of carriers drops rapidly to the order of picoseconds. The corresponding PL decay curves are fitted with bi‐exponential functions (Figure 5d), and the fitting parameters are summarized in Table S3 (Supporting Information). Meanwhile, the dynamical PL intensity (I_max_) increases non‐linearly with the increasing pumping density, which is fitted well by theoretical model. (Figure 5e and Supplementary Note 1). And the curve of radiative time (t_r_, the full‐width at half‐maximum of the dynamical PL peak) decreasing with increasing pumping fluence is plotted in Figure 5f, in line with theoretical simulation as well. Notably, CESF can attain a great shorter decay time (3 ps) than that of SF owing to radiative enhancement by coupling with cavity light filed. And the decay rate of SF is much faster than that of spontaneous radiation in individual QDs (Figure S15, Supporting Information). This is because the collective emission of coupled excitons accelerates the radiation decay rate, and the decay lifetime is inversely proportional to the number of excitons involved in coupling. Hence, we can conclude that the ultrafast optical characteristics of blue CESF are derived from the synergistic effects of synchronous excitons and cavity photons. Furthermore, the long exciton dephasing time of CsPbBr_2_Cl QDs^[^ ^13^, ^51^ ^]^ enables more excitons to participate in the establishment of a coherent cooperative emission process,^[^ ^10^, ^52^ ^]^ contributing to the realization of ultrafast blue CESF.

## 3 Conclusion

In summary, we successfully synthesized well‐defined CsPbBr_2_Cl QDs self‐assembled superlattices and demonstrated that compared to individual QDs, self‐assembled CsPbBr_2_Cl QD superlattice structures can alleviate photon‐induced halide phase segregation through weakening EP coupling. In combination of theoretical analysis and experimental results, the underlying mechanism was clarified. The QD superlattice structure, benefitting from excitonic delocalization, alleviates EP coupling and reduces lattice strain, thereby strengthening the energy barriers of phase segregation. Consequently, ultrafast (3 ps) blue CESF was realized based on the obtained CsPbBr_2_Cl QD superlattices with regularly geometrical structure, in which the densely packed QD arrays are strongly coupled to the self‐formed microcavity. Furthermore, the ultrafast optical property of blue CESF was analyzed by TRPL, and the dynamics of blue CESF was revealed. Additionally, we revealed the ultrafast radiation rate of blue CESF originates from combination of collective cooperative emission behaves and optical cavity coupling, which is facilitated by the long exciton dephasing time of CsPbBr_2_Cl QD superlattices. Our work could shed light on strategies to mitigate halide phase segregation, and the obtained blue CESF with excellent optical properties exhibits promising application prospects in optical communication, ultrafast coherent light sources, displays, and other fields.

## 4 Experimental Section

### Preparation of CsPbBr_2_Cl QDs Superlattices‐Chemicals and Materials

Cesium carbonate (Cs_2_CO_3_, 99.99%), lead chloride (PbCl_2_, 99.99%), lead bromide (PbBr_2_, 99.99%), trioctylphosphine (TOP, 90%), oleic acid (OA, 90%), octylamine (OLA, 90%), oleylamine (OAm, 90%), 1‐octadecene (ODE, 90%), methyl acetate (C_3_H_6_O_2_, 99.5%, anhydrous), and toluene (95%, anhydrous) were purchased from Sigma–Aldrich.

### Preparation of CsPbBr_2_Cl QDs Superlattices‐Synthesis of CsPbBr_2_Cl QDs

CsPbBr_2_Cl QDs were synthesized through modified previous method. In brief, Cs‐oleate precursors were prepared as follows. 200 mg Cs_2_CO_3_, 7.5 ml ODE were loaded into a 50 ml three‐neck flask and dried at 120 °C for 30 min under a N_2_ flow. The Cs‐oleate precursor solution was maintained at 100 °C to prevent its precipitation out of ODE. In another 100 mL three‐neck flask, 73 mg PbBr_2_, 56 mg PbCl_2_ and 1 ml TOP were suspended in 10 ml ODE, dried at 120 °C for 30 min under a N_2_ flow. Afterward, 1 ml OAm, 1 ml OLA and 0.1 ml OA were injected into the flask under N_2_ flow. After PbCl_2_ and PbBr_2_ completely dissolved, the reaction mixture was heated up to 175 °C then down to 170 °C and 1 ml Cs‐oleate was swiftly injected. After 5 s, the reaction mixture was cooled down in an ice‐water bath, followed by centrifugation to collect the solid products. Thereafter, the products were redispersed in toluene and centrifuged again and the precipitate was discarded. The supernatant was destabilized by adding methyl acetate, followed by centrifuging and dispersing the QDs in toluene for further use.

### Preparation of CsPbBr_2_Cl QDs Superlattices‐Self‐Assembly of CsPbBr_2_Cl QDs into Superlattices

The as‐prepared QDs were kept in a dark and vibration‐free environment at ≈12 °C for 6–10 days for low temperature aging. Then 20 µL CsPbBr_2_Cl QD solution was dropped on a 15 mm × 15 mm clean silicon wafer in a saturated toluene vapor atmosphere for slow solvent evaporation. CsPbBr_2_Cl superlattices were formed on the silicon substrate.

### Structural, Optical Characterizations, and TRPL Characterizations

TEM analysis was performed using a transmission electron microscope (HEOL‐2010). The samples were previously dropped on a clean wafer and then transferred onto a 300‐mesh copper TEM grid by spot cleaning. SEM images were collected via field‐emission scanning electron microscopy (FE‐SEM; Auriga S40, Zeiss, Oberkochen, Germany). The crystal structures of the prepared samples were identified by the X‐ray diffraction (XRD) with Cu‐K*α* radiation (PANalytical Empyrean). The samples were put in a Dewar (77–475 K, Janis ST‐500) with a temperature controller (cryocon 22C) for temperature‐dependent PL experiments. The excitation source was a 400 nm femtosecond (fs) laser (Libra, Coherent, ≈40 fs, 10 kHz). The PL spectra were obtained by a 50 × microscopy objective (NA = 0.50) in a confocal fluorescence detection system (LabRAM HR Evolution). The spectral resolution of Horiba Raman spectroscopy is ≈0.0166 nm. The dynamical measurements were performed on a streak camera (C10910‐05, M10911‐01) with a closed‐cycle high‐vacuum dewar (MONTANA) at 77 K.

### DFT Calculations

In the computational method, electronic structure calculations were performed in the framework of DFT. Detailed information is available in Supporting Information.

## Conflict of Interest

The authors declare no conflict of interest.

## Supporting information

Supporting Information

## Data Availability

The data that support the findings of this study are available from the corresponding author upon reasonable request.
