# Supplementary material for: Stable and Ultrafast Blue Cavity‐Enhanced Superfluorescence in Mixed Halide Perovskites
Source: Adv Sci (Weinh). 2023 May 1;10(21):2301589. doi: 10.1002/advs.202301589 (PMC10375166; doi:10.1002/advs.202301589)
Supplement: Supplementary file 1 — Supporting Information [file ADVS-10-2301589-s001.pdf]

ADVANCED SCIENCE
Open Access

Supporting Information

for *Adv. Sci.*, DOI 10.1002/advs.202301589

Stable and Ultrafast Blue Cavity-Enhanced Superfluorescence in Mixed Halide Perovskites

Linqi Chen, Danqun Mao, Yingjie Hu, Hongxing Dong, Yichi Zhong, Wei Xie*, Nanli Mou, Xinjie Li and Long Zhang**

Supporting information for

Stable and ultrafast blue cavity-enhanced superfluorescence in mixed halide perovskites

Linqi Chen[⊥§∥], *Danqun Mao*[◊∥], *Hongxing Dong*[⊥†Ɨ*], *Yichi Zhong*[Ɨ], *Wei Xie*[◊*], *Nanli Mou*[†], *Xinjie Li*[⊥], *Long Zhang*[⊥†Ɨ*]

⊥Key Laboratory of Materials for High-Power Laser, Shanghai Institute of Optics and Fine Mechanics, Chinese Academy of Sciences, Shanghai, 201800, China.

†Hangzhou Institute for Advanced Study, University of Chinese Academy of Sciences, No.1, Sub-Lane Xiangshan, Xihu District, Hangzhou 310024, China.

§Center of Materials Science and Optoelectronics Engineering, University of Chinese Academy of Sciences, Beijing, 100049, China.

◊ State Key Laboratory of Precision Spectroscopy, School of Physics and Electronic Science, East China Normal University, Shanghai 200241, China.

Ɨ CAS Center for Excellence in Ultra-intense Laser Science, Shanghai, 201800, China.

DFT calculations

PBE0 Polaron

Density function theory calculation were performed by using the CP2K package. Unrestricted Kohn-Sham DFT has been used as the electronic structure method in the framework of the Gaussian and plane waves method. The Goedecker-Teter-Hutter (GTH) pseudopotentials, DZVP-MOLOPT-GTH basis sets were utilized to describe the molecules. A plane-wave energy cut-off of 500 Ry has been employed. To accurately model the polaron, PBE0 functional with Grimme D3 correction was used to describe the system.

$$E_{xc}^{PBE0} = 1/4E_x^{HF} + 3/4E_x^{PBE} + E_c^{PBE}$$

The faction of Hartree-Fock exchange is set to 0.25. Due to the high cost of standard hybrid functional, the Auxiliary Density Matrix Method (ADMM) has been used. The bulk material is simulated using within a cubic box with a dimension of 23.75 × 24.02 × 23.75 Angstrom, which contains 320 atoms. The electron polaron is modelled with one excess electron in the bulk material. To stabilized the polaron, we performed at 300 K using Canonical sampling through velocity rescaling with the time step of 2 fs. Once the polaron is trapped by the material, we further perform geometry optimization to obtain its stationary state.

Anion migration path and energy barrier

Density functional theory (DFT) calculations were performed with CASTEP package on the basis of the plane-wave-pseudo-potential approach. The interactions between the atomic core and the valence electrons were described by the normconserving pseudopotential for energy band calculations and ultrasoft pseudopotential for the transition states search, respectively. The migration of Br(Cl) vacancy was investigated by searching the possible migration route and identifying the migration transition state with the lowest diffusion energy barrier. We assumed that the migration of Br(Cl) vacancy is the self-diffusion from one defective lattice site to its nearest neighboring Br(Cl)site. The migration energy barrier is the energy difference between the total energies of transition state and the initial defective structure. The transition state is searched by the generalized synchronous transit (LST/QST) method

implemented in the CASTEP code.

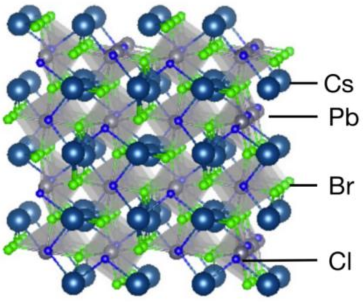

Figure S1. 3D-perovskite orthorhombic crystal structure (Pnma space group) of $CsPbBr_2Cl$.

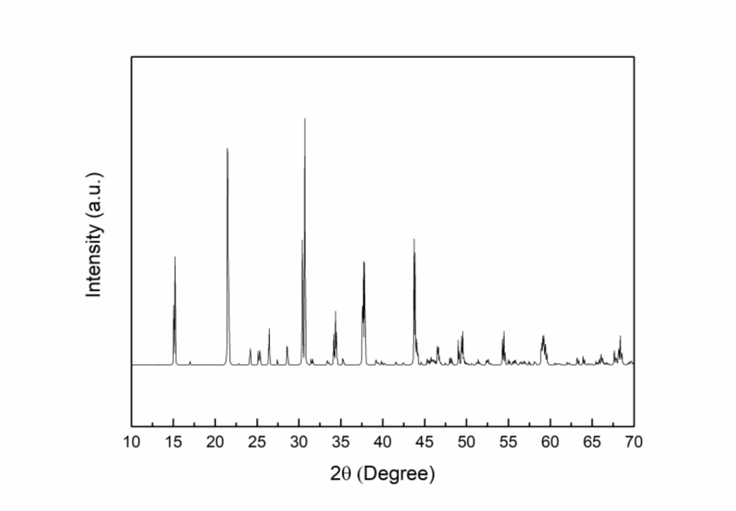

Figure S2. Theoretical simulation of the XRD pattern of 3D-perovskite orthorhombic $CsPbBr_2Cl$.

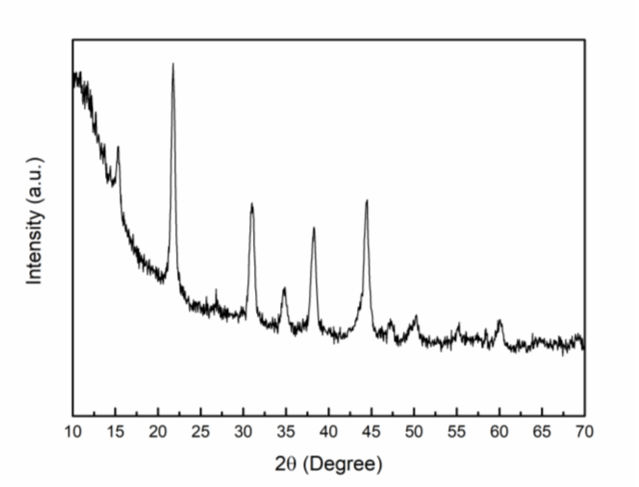

Figure S3. X-ray diffraction results of $CsPbBr_2Cl$ QDs.

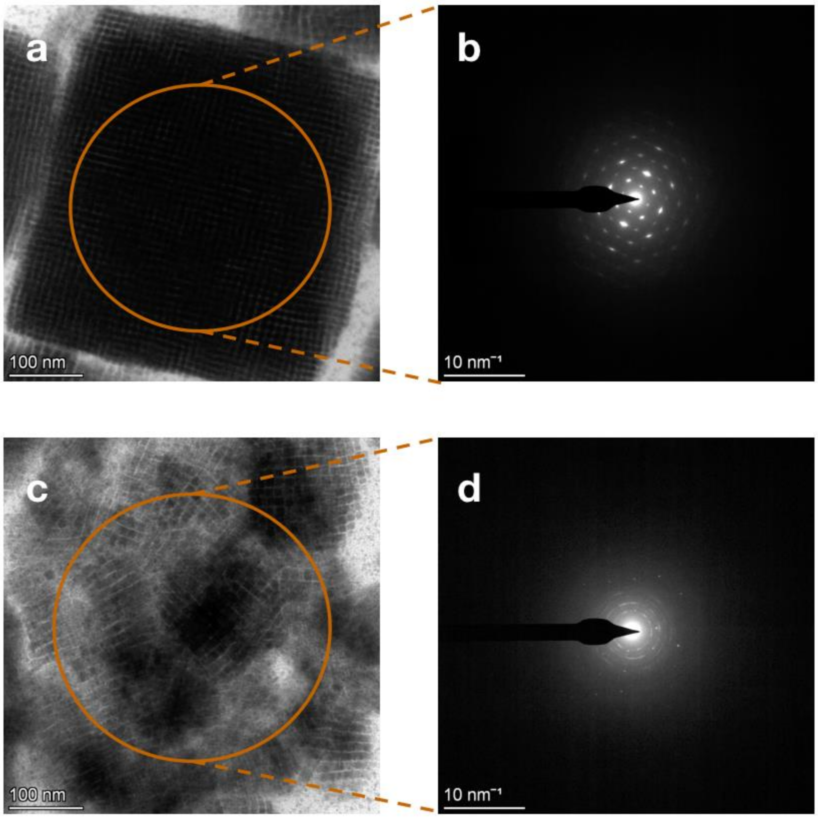

Figure S4. (a) TEM image of $CsPbBr_2Cl$ QD superlattice structure. (b) The selected electron diffraction pattern of orange circle part in (a). (c) TEM image of low-ordered $CsPbBr_2Cl$ QD clusters. (d) The selected electron diffraction pattern of orange circle part in (c).

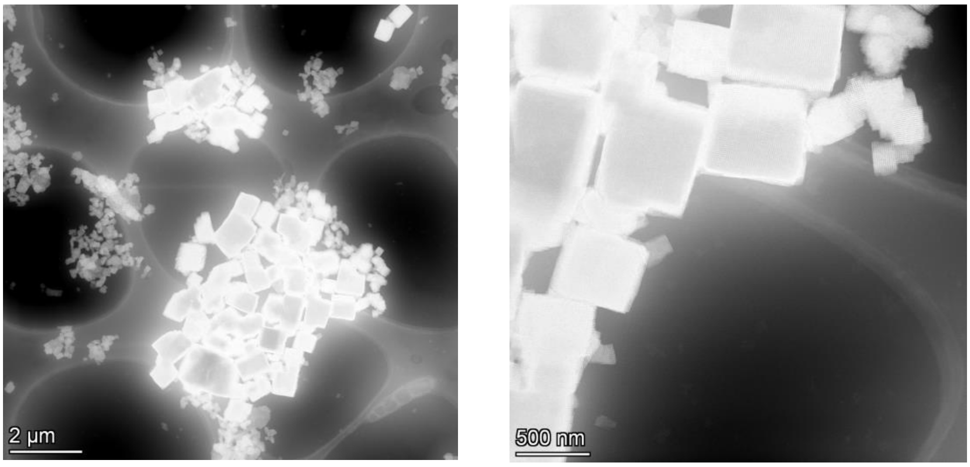

Figure S5. large area TEM images of $CsPbBr_2Cl$ superlattices.

a

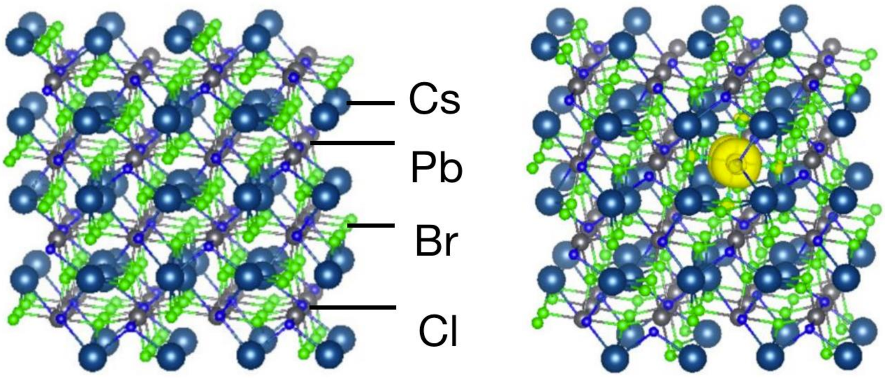

b

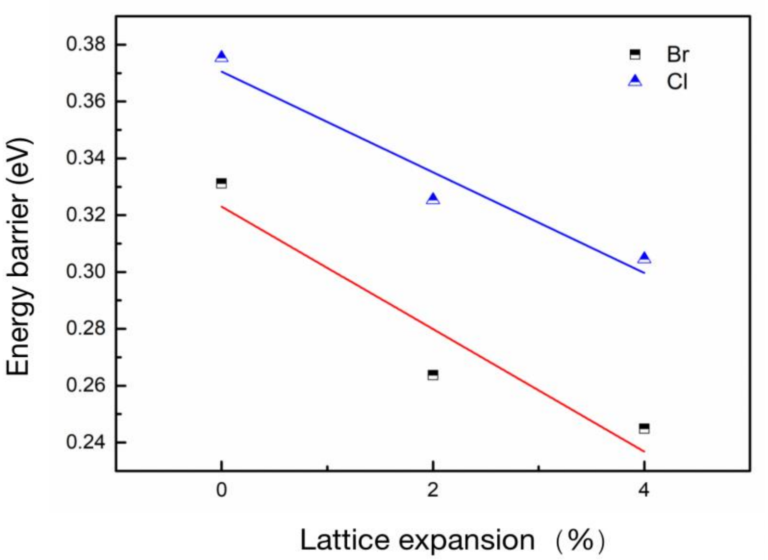

Figure S6. (a) The lattice structure change of $CsPbBr_2Cl$ before and after the generation of polaron. (b) Calculated activation energy for Cl and Br diffusion in $CsPbBr_2Cl$ as a function of lattice expansion.

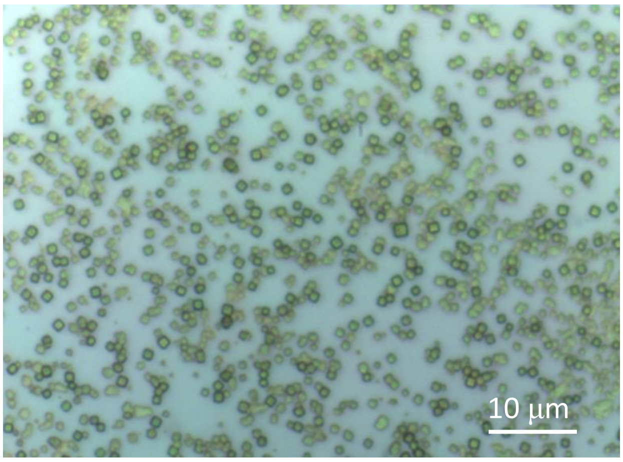

Figure S7. Large area microscope images of $CsPbBr_2Cl$ SLs.

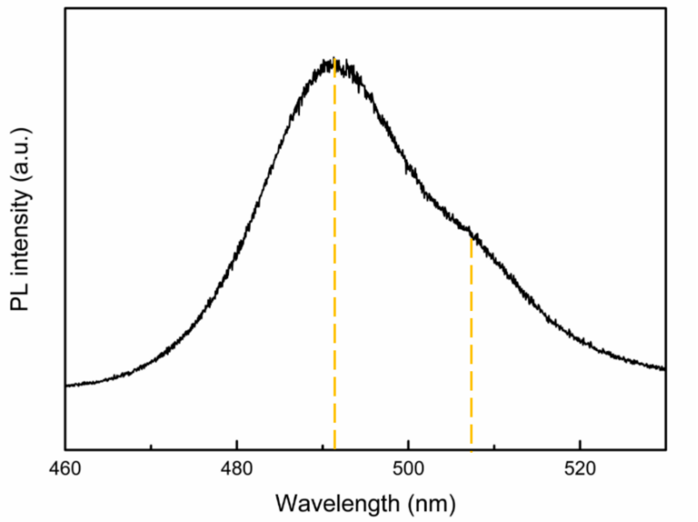

Figure S8. PL spectrum of $CsPbBr_2Cl$ superlattices under CW irradiation of 147 W cm^{-2} after 60 min.

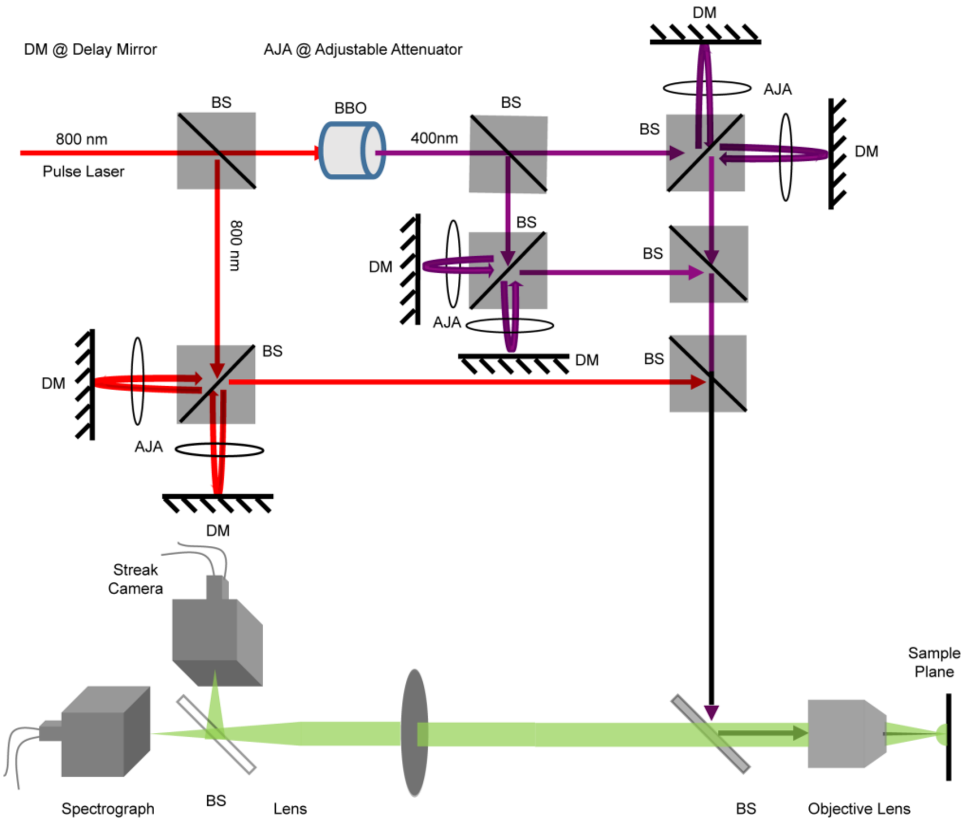

Figure S9. The diagram of the self-built confocal microscope photoluminescence system.

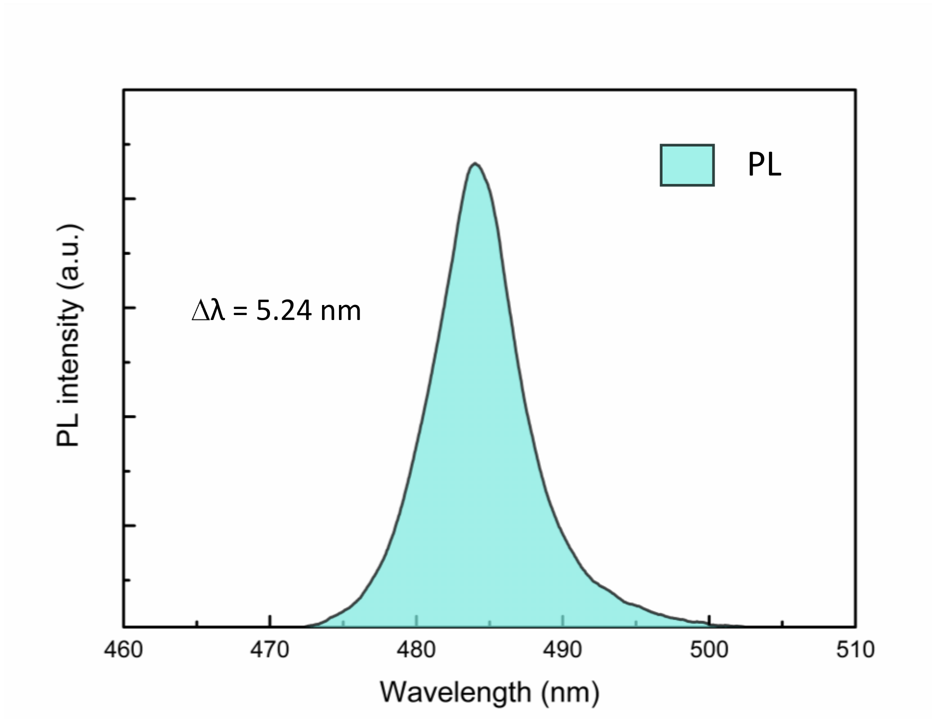

Figure S10. PL spectrum of QDs superlattice below threshold (SF) at low temperature, and the corresponding FWHM is 5.24 nm.

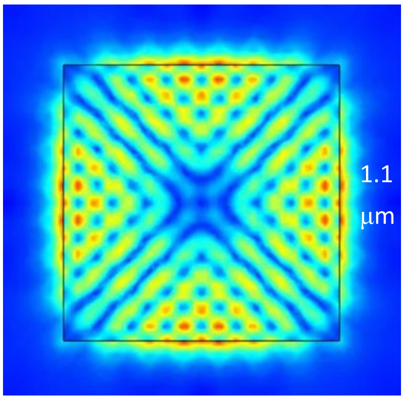

Figure S11. Simulated 2D normalized enectric field of λ~486.5 nm of $CsPbBr_2Cl$ quantum dot superlattice, defining a WGM standing-wave cavity mode.

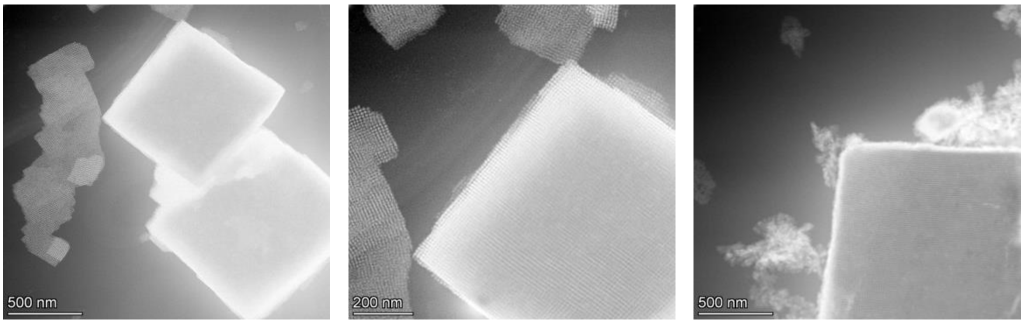

Figure S12. TEM images of QD superlattices on Si substrate kept for one month.

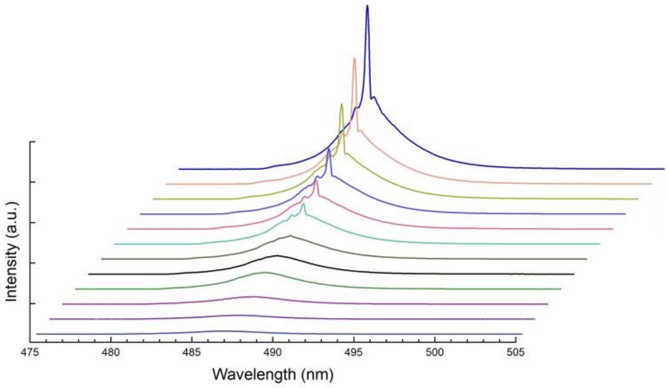

Figure S13. Power-dependent emission spectra of superlattice kept for one month.

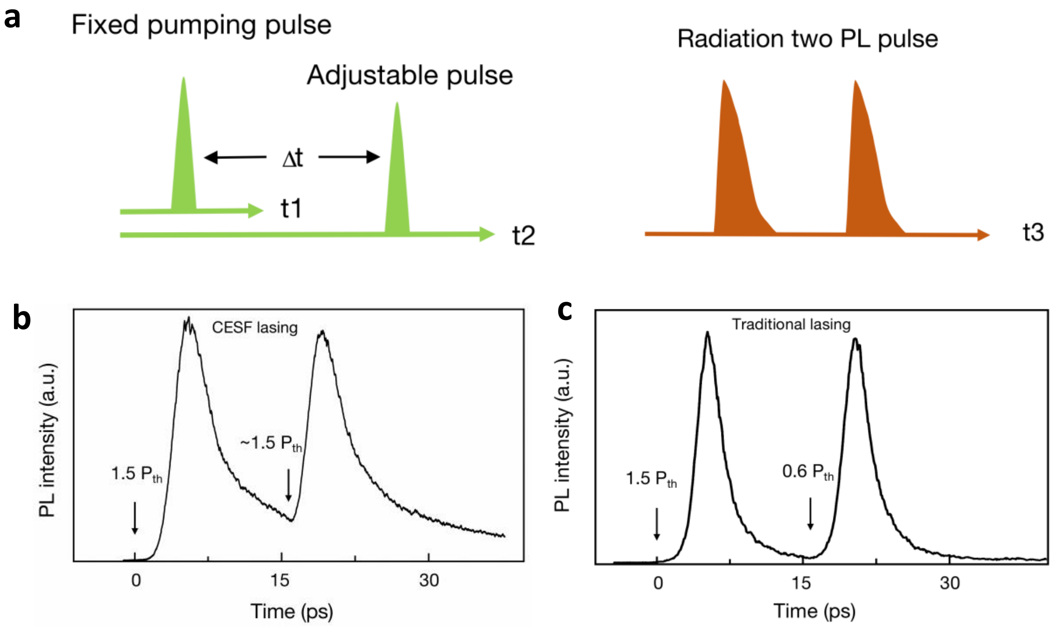

Figure S14. Schematic diagram of the experimental method. The two femtosecond laser pulses excite the sample in a time interval of Δt. In the experiment, we chose the intensity of the 1.5 P_{th} as the reference value. Then, the intensity of the second excitation light is adjusted to make the intensity of the two radiated PL consistent. (b) Time dynamic CESF spectra based on $CsPbBr_2Cl$ perovskite QDs superlattice pumping by two laser pulses. (c) Time dynamic lasing spectra based on $CsPbBr_2Cl$ perovskite microspheres. For CESF, to reach the same PL intensity of two times of radiation, the energy of the first excitation is similar to the energy of the second excitation, indicating that most of the carriers in the sample have been exhausted after the first radiation. For PL lasing, it needs to meet population inversion condition. After the stimulated emission is completed quickly, there are still a lot of remaining carriers that emit slower spontaneous emission. So the energy of the second excitation is much lower than the first time.

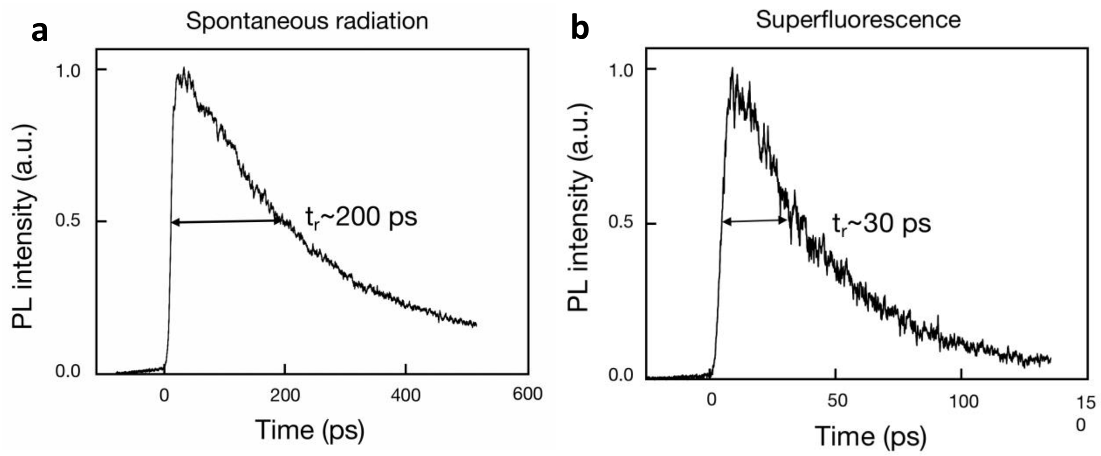

Figure S15. Monodisperse QDs spontaneous radiation with a radiative time t_r (FWHM) of ~200 ps, while self-cooperating excitons in the SL emit a SF pilse with t_r of ~30 ps.

Table S1. Fitting results of temperature-dependent PL peak energy.

Sample	E_0 (eV)	A_T (meV)	A_{EP} (meV)	$\hbar\omega$ (meV)
SLs	2.665	0.061	130.7	175.1
QDs	2.7	0.238	151.8	133.9

Table S2. Fitting results of temperature-dependent FWHM of PL peak.

Sample	Γ_{inh} (meV)	Γ_{AC} (μeV K^{-1})	Γ_{LO} (meV)	E_{LO} (meV)
SLs	28.4	50.2	15.5	21.4
QDs	36.9	81.0	30.6	28.9

Table S3. Fitting parameters of PL decay curves under different pumping density.

Excitation density	τ_1 (ps)	$A_1/(A_1+A_2)$ (%)	τ_2 (ps)	$A_2/(A_1+A_2)$ (%)
0.7 P_{th}	58.5	100	-	-
1.1 P_{th}	18.5	48.3	57.4	51.7
1.8 P_{th}	3.1	94.2	37.8	5.8

Supplementary Note 1: Theoretical Description

Theoretical Model: Dicke Model in a cavity

Based on the *Dicke* model, we study the radiation of cooperative excitons in our samples[1,2]. Here, the excitons in QDs are simplified as the ideal dipoles in a two level system. The inhomogeneous broadening of QDs is ignored. For the free Hamiltonian of a two-level dipole we have,

$$\hat{H}_1 = \begin{pmatrix} \frac{1}{2}\hbar\omega_0 & 0 \\ 0 & -\frac{1}{2}\hbar\omega_0 \end{pmatrix} \tag{1}$$

where the zero energy has been chosen at the mid-point of the energy interval $\hbar\omega_0 = E_e - E_g$. The dipole moment operator $\hat{d}$ can be written as an off-diagonal matrix,

$$\hat{d} = \begin{pmatrix} 0 & d_{eg} \\ d_{ge} & 0 \end{pmatrix} \tag{2}$$

All the two-level Hermitian operators can be expressed in terms of the Pauli matrices and the unit 2×2 matrix,

$$\hat{\sigma}_1 = \begin{pmatrix} 0 & 1 \\ 1 & 0 \end{pmatrix} \quad \hat{\sigma}_2 = \begin{pmatrix} 0 & -i \\ i & 0 \end{pmatrix} \quad \hat{\sigma}_3 = \begin{pmatrix} 1 & 0 \\ 0 & -1 \end{pmatrix} \quad \hat{\sigma}_0 = \begin{pmatrix} 1 & 0 \\ 0 & 1 \end{pmatrix} \tag{3}$$

In particular, in accordance with (1) and (2)

$$\hat{H}_1 = \frac{1}{2}\hbar\omega_0\hat{\sigma}_3 \qquad \hat{d} = \mathrm{Re}\,d\,\hat{\sigma}_1 + \mathrm{Im}\,d\,\hat{\sigma}_2 \tag{4}$$

where $\mathrm{Re}\,d$ and $\mathrm{Im}\,d$ are the corresponding real and imaginary parts of d_{ge} •

Let us introduce the quasi-spin operators for the i th dipole

$$\hat{R}_1^{(i)} = \frac{1}{2}\hat{\sigma}_1^{(i)} \qquad \hat{R}_2^{(i)} = \frac{1}{2}\hat{\sigma}_2^{i(} \qquad \hat{R}_3^{(i)} = \frac{1}{2}\hat{\sigma}_3^{i(} \tag{5}$$

and the total quasi-spin operators of the *N*-dipole system

$$\hat{R}_\alpha = \sum_{i=1}^{N}\hat{R}_\alpha^{(i)} \qquad \alpha = 1,\ 2\,, \tag{6}$$

which obey the usual commutation rules of angular momentum operators

$$\left[\hat{R}_\alpha^{(k)}, \hat{R}_\beta^{(j)}\right] = i\delta_{kj}\hat{R}_\gamma^{(k)} \qquad \left[\hat{R}_\alpha, \hat{R}_\beta\right] = i\ \hat{R} \tag{7}$$

Where α, β, γ is any cyclic permutation of the numbers $1, 2, 3$. Then the energy operator of an ensemble of N free identical two-level dipoles can be expressed in the form

$$\hat{H}_N = \sum_{i=1}^{N} \hbar\omega_0 \hat{R}_3^{(i)} = \hbar\omega_0 \hat{R}_3 \tag{8}$$

And its eigenvalues are equal to $\hbar\omega_0 M$, where $M = \frac{1}{2}N, \frac{1}{2}N - 1,, -\frac{1}{2}N$ is the eigenvalue of $\hat{R}_3$.

The Hamiltonian for the interaction of such an N-dipole system with the electromagnetic field can be chosen to be in the form

$$\hat{H}_{int} = -\hat{\varepsilon}\sum_{i=1}^{N} \hat{d}^{(i)} = -\hat{\varepsilon}\, d \sum_{i=1}^{N} 2\hat{R}_1^{(i)} \tag{9}$$

where $\hat{\varepsilon}$ is the operator of the electric field, $\hat{d}^{(i)}$ is the electric dipole moment operator of the ith dipole, $\hat{d}^{(i)} = 2d\hat{R}_1^{(i)}$.

All dipoles are initially in their excited states. The radiative decay of such an ensemble is a cascade of transitions between the adjacent states with the same eigenvalue of $\hat{R}^2$ equal to $\frac{1}{2}N(\frac{1}{2}N+1)$

$$\left|\frac{1}{2}N, \frac{1}{2}N\right\rangle \to \left|\frac{1}{2}N, \frac{1}{2}N - 1\right\rangle \to \cdots \to \left|\frac{1}{2}N, -\frac{1}{2}N\right\rangle \tag{10}$$

The probability per unit time, $\gamma_{M,M-1}$ for a transition $M \to M$-1, can be obtained,

$$\gamma_{M,M-1} = \gamma(\frac{1}{2}N + M)(\frac{1}{2}N - M + 1) \tag{11}$$

For the SL samples with cavity effect:

When the cooperative dipoles are placed into an ***optical cavity***, the parameter γ in equation (11) needs to ***enlarge by a factor N$_p$***, i.e., the effective photon number coupling with dipoles, or the amplification factor of optical field density between the cases 'with cavity' and 'without cavity'. And ***we need to add a new equation into the rate equations (12) to describe the dynamics of*** N_p,

$$\frac{dN_P}{dt} = \bar{I}(t) - N_P / t_c \tag{14}$$

Here, t_c is the photon lifetime of optical cavity. In addition, a filling factor f describes the ratio between the number N of available hosts for cooperative dipoles and the initially excited dipole number N_0 in the system.

We use the following parameters to obtain the numerical solutions, the excited dipole number by pumping in a cooperative volume $N_0 \in [0\sim50]$, the filling factor $f = 3$, the spontaneous radiative rate $\gamma = 0.004\ \mathrm{ps}^{-1}$, the cavity photon lifetime $t_C = 2\ \mathrm{ps}$. The theoretical results are plotted in **Fig. 5e,f**.
